# Joint inference of ancestry and genotypes of parents from children

**DOI:** 10.1016/j.isci.2022.104768

**Published:** 2022-07-16

**Authors:** Yiming Zhang, Yufeng Wu

**Affiliations:** 1 Department of Computer Science and Engineering, University of Connecticut, 371 Fairfield Way, Unit 4155, Storrs, CT 06269-4155, USA

**Keywords:** Genomics, Bioinformatics, Algorithms

## Abstract

In this paper, we address a problem: can we perform ancestry inference for parents from one or more children’s DNA samples? That is, suppose the parents’ genomes consist of segments of different ancestry, and our goal is inferring parental ancestry and at the same time, calling parental genotypes from given children’s genetic data. Such ancestry inference may provide insights into recent ancestors from children’s genomes, and potentially has applications in understanding genetic traits. At present, there exists no method for this inference problem. We present parMix, a method based on hidden Markov model (HMM) that can jointly infer parental ancestry and call parental genotypes from data of a small number of children. Simulation results show that parMix performs well in practice. It can provide reasonably accurate parental inference given data from a small number (say three) of children. parMix becomes more accurate when data from more children are used.

## Highlights

• Presented a method for inferring ancestry and genotypes of parents from children
• Recombination events can be detected using parMix
• parMix can deal with the genotypes with phasing errors
• parMix can be used to infer admixture proportion of parents

## Graphical abstract

**Figure:**
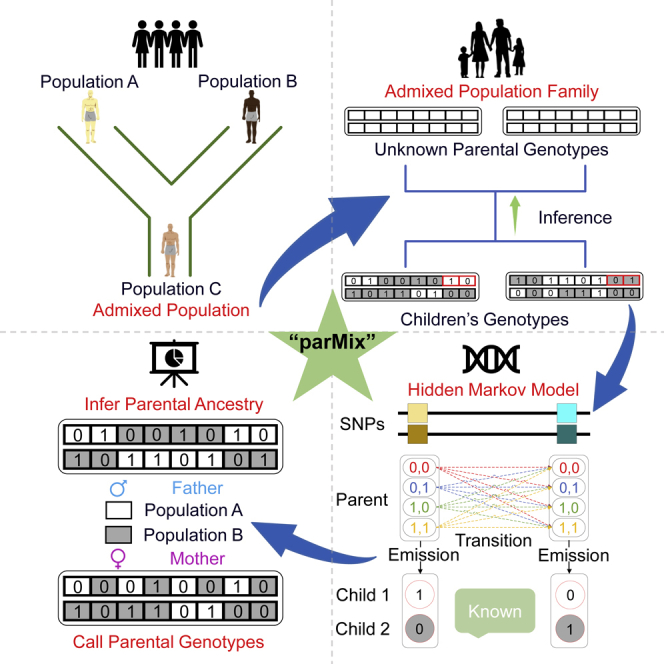

## Introduction

Geneticists have envisioned using genetic tests to infer something about ancestors long ago (Doolittle 1981; Royal et al., 2010). Nowadays, DNA ancestry tests offered by companies such as Ancestry.com or 23andMe let people know not only something about themselves but also more about their ancestors. Existing ancestry tests often concern the genomic ancestry composition of the focal individual (i.e., who provides the DNA to test). It is widely believed that population admixture is widespread in human populations (Pritchard et al., 2000; Price et al., 2007; Maples et al.) So focal individuals (and their recent ancestors) are likely admixed. The admixture tests are often referred to as “chromosome painting” (Pritchard et al. 2000; Alexander et al. 2009; Sankararaman et al., 2008; Price et al., 2009), where the genome of the focal individual is broken into segments and these segments originate from different ancestral populations.

Existing chromosome painting methods usually implicitly assume that two parents have the same ancestry composition (Pritchard et al. 2000). This is arguably unrealistic because it is possible that the two parents may have different ancestry themselves. For example, suppose a focal individual has a DNA composition of 50% from population A and 50% from population B. Then the DNA composition of the two parents of this individual may be (among other possibilities): (i) both are 50% from A and 50% from B, or (ii) one is 100% from A and the other is 100% from B. Therefore, a natural research question is can we infer the genetic ancestry composition of our recent ancestors, such as parents, from focal individuals’ DNA?

PedMix (Pei et al., 2020) is one of the first methods for recent ancestry inference. It can infer ancestry proportions of parents or grandparents of the focal individual from a single individual’s genotypes. However, it has a major drawback: it only provides an estimate of ancestry proportions but cannot perform more fine-scale inference of ancestry of recent ancestors. Ideally, one may want to perform chromosome painting for recent ancestors from the genotypes of a focal individual. It is also useful to call parental genotypes (not performed by PedMix). This kind of parental ancestry inference can lead to more insights into the genetic composition of parents than their average genetic composition, and can be potentially useful in studying questions, e.g., understanding genetic traits.

Chromosome painting of parents and parental genotype calling with only genotypes from a single child, while possible, is not easy. Consider the case of parents. The child only has 50% of DNA from each parent. That is, 50% of parental DNA is missing. Technical difficulties such as phasing errors further complicate the inference. There are few existing methods that can perform ancestry inference in this setting. Existing methods for parental genotype calling are mainly developed for animal breeding, which usually involves a large number of offspring. LSPH (Baruch et al., 2006) attempts to recover the missing data of parents’ haplotypes from the offspring’s genotypes. However, LSPH assumes parents are not admixed and does not perform inference of ancestry. Note that a single child may not provide sufficient information about two parents. To develop a practical inference method, it may be useful to use genetic data from two or more children. Genetic data from multiple children of the same two parents may together allow more accurate inference. cnF2freq (Nettelblad 2012) uses hidden Markov models (HMMs) to reconstruct the genotypes of individuals in a full sib-ships pedigree that contains a large number (say 20) of children. But it does not consider the situation that parents are admixed.

In this paper, we develop methods for inferring both ancestry and genotypes of parents of a small number of children. Our method is implemented in a computer program called “parMix”, which is available for download at https://github.com/biotoolscoders/parmix. parMix takes genotypes from multiple children and population genetic information (e.g., allele frequencies of ancestral populations) as input. parMix can infer parental ancestry and call parental genotypes at each single nucleotide polymorphisms (SNPs). That is, different from PedMix, parMix can perform chromosome painting of parents and call parental genotypes from children’s genotypes, instead of just estimating the overall ancestry proportions. To the best of our knowledge, there are no existing methods that infer both ancestry and genotypes of parents from a small number (say two) of children’s genotypes. Simulation results show that parMix performs reasonably well in parental ancestry inference and genotype calling.

## Results

### Results on simulated data

#### Simulation

Table 1 shows the parameters (with explanation and the default values) that we use in the simulations. We first simulate $n_{h}$ haplotypes using *macs* (Chen et al. 2009) from two ancestral populations which diverged from one ancestral population at $4N_{e}t$ generations in the past. Then, an admixed population is formed by merging the two ancestral populations and simulating *forward* in time the process of random mating, genetic drift, and recombination using a diploid Wright-Fisher model for *g* additional generations. Finally, we randomly select $4n_{f}$ haplotypes to form $n_{f}$ families and do the simulation for one additional generation. This leads to $n_{k}$ children per family. The recombination rate variation from the 1000 Genomes Project (The 1000 Genomes Project Consortium, 2015) is used. The default length of the chromosome is $2.59\times 10^{8}$ bps, which is the length of the first chromosome of humans. Haplotypes are paired to create genotypes. Phasing errors are then added stochastically by switching between two parental chromosomes according to a Poisson process with a rate $p_{p}$. The number of SNPs for one chromosome simulated by macs is ∼0.13M under the default settings. Processing data with this size can be slow. Thus, we perform a frequency-based pruning method (Pei et al., 2020) to trim data. This frequency-based pruning approach removes SNPs with a minor allele frequency difference in two ancestral populations less than the pruning threshold $d_{f}$. This leaves ∼28,200 SNPs after pruning under the default settings. We perform extensive simulations to evaluate the impact of values of parameters on the inference accuracy of parMix.

**Table 1:** List of parameters and their default values in simulation

Symbol	Default	Description
$n_{h}$	400	Number of haplotypes
$n_{c}$	1	Number of chromosomes
$N_{e}$	10,000	Effective population size
*L*	$2.59\times 10^{8}$	Region length (bp)
μ	$1\times 10^{-9}$	Mutation rate (per generation per bp)
ρ	$1\times 10^{-8}$	Recombination rate (per generation per bp)
*t*	0.125	Ancestral populations splitting time
*g*	10	Number of generations since admixture
$n_{f}$	10	Number of families to infer
$n_{k}$	3	Number of kids per family
$d_{f}$	0.1	Frequency-based pruning threshold
$p_{p}$	$2\times 10^{-6}$	Phasing error rate

To evaluate inference accuracy, we compare the called parental genotypes (or ancestry) with the true simulated genotypes (or ancestry) at each locus. Accuracy is measured as the ratio of the number of SNP sites with correct inferred results and the total number of SNP sites. There is one technical issue for comparing inferred parental ancestry and true parental ancestry. The inferred genotypes and ancestry by parMix are from two parental haplotypes. But there is no information about which parent corresponds to a specific inferred result. We use a “best-match” approach for performing the comparison. After the inference, there are four inferred ancestry vectors, one per parental haplotypes; the four haplotypes are grouped for two parents; then the best match results are used among all eight match-ups between the inferred parents and simulated ones.

#### Parental genotype calling and ancestry inference

We first evaluate the performance of parMix under various trimming settings. The results are shown in Table 2. We test different trimming threshold values from 0.05 to 0.5. It can be seen that trimming threshold directly influences the accuracy. For the ancestry inference part, the accuracy usually increases when some non-informative SNPs were discarded with a smaller trimming threshold. When more SNPs are discarded by more aggressive trimming, we start to lose informative SNPs, and accuracy decreases. Then, with more SNPs discarded, the accuracy starts to swing, and the standard deviation increases. However, for the genotypes inference part, since we use LD to infer genotypes, the fewer SNPs were trimmed, the higher the accuracy rate is. Table 2 also shows the running time under different trimming thresholds, where less trimming leads to a longer running time. So, there is a trade-off between inference accuracy and efficiency when choosing trimming threshold.

**Table 2:** Effect of data trimming on accuracy (parental ancestry and genotypes) and efficiency

Threshold	SNPs Remained	Running time (s)	Accuracy rate (A)	Accuracy rate (G)
0.05	41,918	∼35820	79.05%	87.68%
0.1	28,265	∼24070	80.16%	83.76%
0.2	15,049	∼12710	78.69%	80.59%
0.3	8354	∼7050	75.96%	77.08%
0.4	4525	∼3760	78.45%	79.29%
0.5	2293	∼1820	77.44%	77.08%

**Figure 1:**
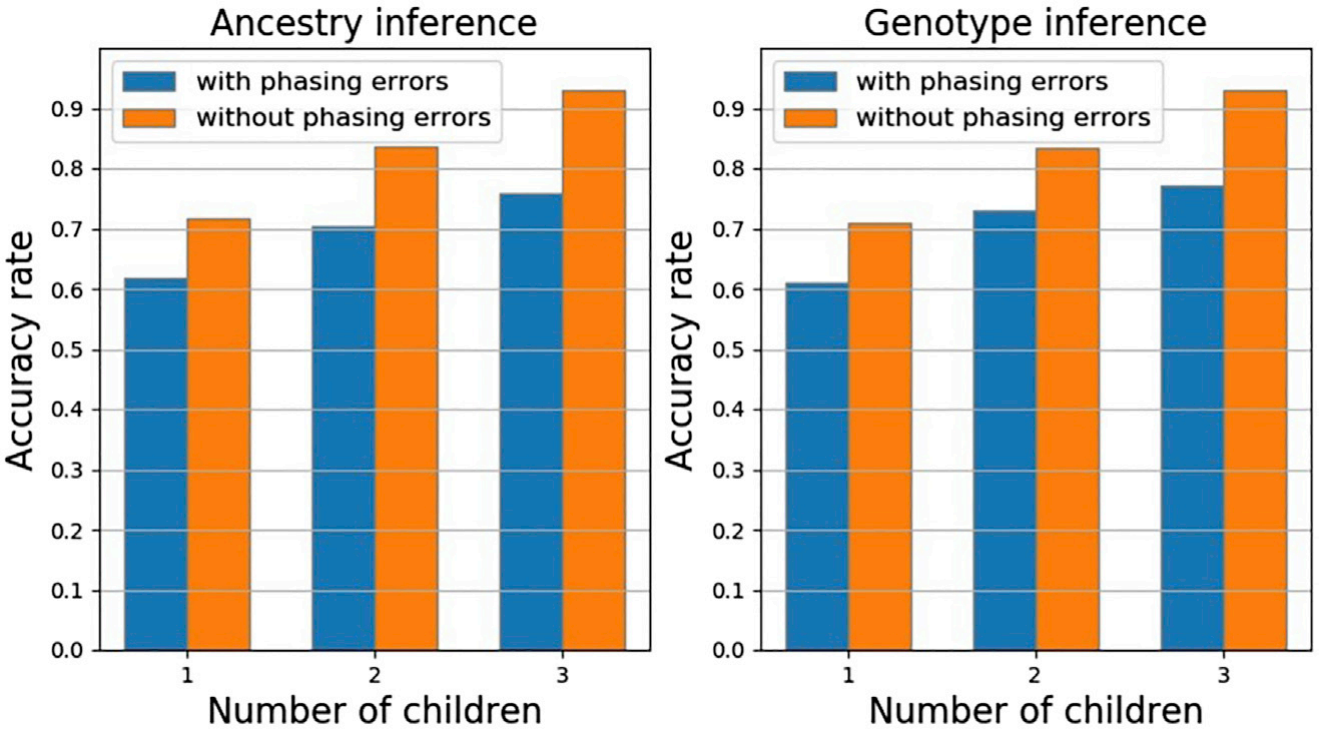
Accuracy of parental genotype calling and ancestry inference with one, two, or three children The trimming threshold is set as 0.3 for all the cases. Two cases: (i) with phasing errors and (ii) without phasing error.

Furthermore, the number of children in a family has a strong influence on inference accuracy. The more children in the family, the higher the accuracy of parMix. The Figure 1 shows this result. A family with only one child leads to a low accuracy rate. We note that even with a single child, genotype/ancestry inference can still be as high as 70% for the case of no phasing errors, which is much higher than random guess. Parental inference accuracy steadily increases with the addition of more children. With three children, for example, parental inference accuracy can have over 93% accuracy without phasing error. Even with phasing errors, parMix still achieves close to 80% accuracy. This indicates that parMix can indeed be useful for parental inference. Note that with more children, the computational time of parMix increases.

#### Comparison of parMix with other methods

To evaluate the performance of parMix, we compare it with PedMix. PedMix is used to estimate the parental admixture proportions as the average of the admixture proportions inferred from haplotypes of each child *independently*. We then run parMix to infer the parental ancestry, and calculate admixture proportion based on the inferred ancestry. Note that parMix analyzes all children together which can extract more information about the joint parental history. We run both PedMix and parMix on haplotypes from three children. For PedMix, we pre-processed the haplotypes with trimming threshold as 0.3, and phasing errors with rate $p_{p}=0.000002$ per bp. In addition, we use a single SNP calling method as the baseline of inference. This method uses given allele frequencies of two ancestral populations and children’s genotypes at each SNP site. Then, it infers the genotypes and ancestry information of parents. If the SNPs of children are all 0 (resp. 1) on the same locus, the parents’ genotype at this position is set to 00 (resp. 11). If 0 and 1 are both present in children’s alleles, the parents’ genotype is set to 01 or 10 arbitrarily. Then, the ancestries of parents are also inferred based on the called genotypes from the previous step and allele frequencies of ancestral populations. The ancestral population with higher allele frequency is chosen as the ancestry on this position.

**Figure 2:**
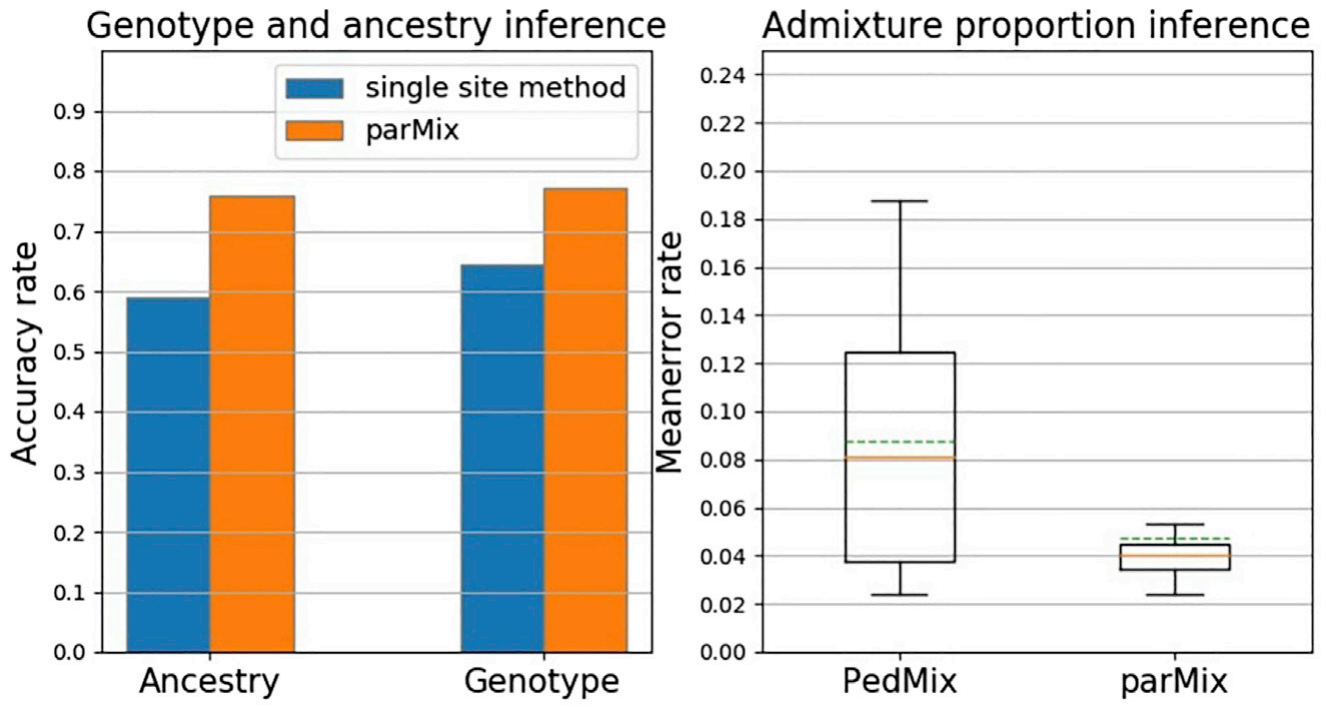
Comparison of parMix with other methods Two cases: (i) parMix vs. single site method. (ii) parMix vs. PedMix. parMix has much lower error rate than PedMix.

Figure 2 (i) shows the accuracy of parMix and the single site inference method (as explained above). As expected, parMix is clearly better than the above single site inference method. Note that single site inference is not a random guess: a random guess is expected to have accuracy much lower than 50% for parental genotype calling. Moreover, Figure 2 (ii) shows that parMix outperforms PedMix in parental admixture proportion estimate with three children. This is because parMix uses more information from data than PedMix. Also note that PedMix can infer more distant ancestors, e.g., grandparents.

#### Phasing error

parMix requires the phasing error rate as an input. In practice, it may not be easy to know the exact phasing error rate. To evaluate the impact of the misspecified phasing error parameter, we run the parMix by specifying data with a different phasing error parameter, which is ten times of the true phasing error rate. We also investigate whether phasing error correction can improve the accuracy. For this, we use the phasing errors correction method developed in PedMix.

**Figure 3:**
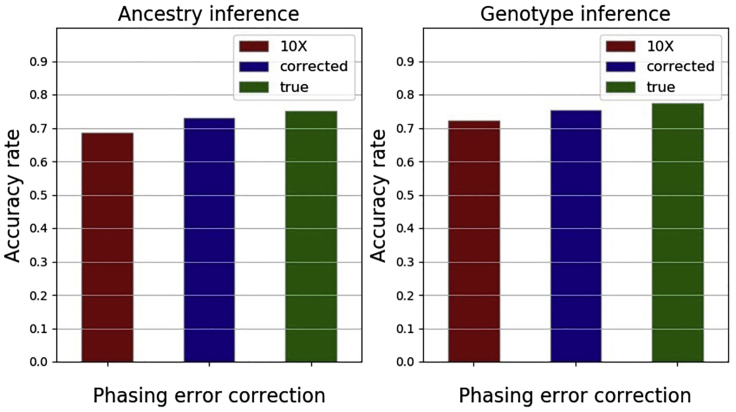
Impact of phasing error (i) left bars: mis-specification of phasing error rate slightly reduces accuracy, (ii) middle bars: correcting phasing errors can improve accuracy, and (iii) right bars: using the correct phasing error rate leads to more accurate inference.

As shown in Figure 3, with trimming threshold as 0.3, parental inference accuracy reduces when the phasing error rate is mis-specified (to be 10 times as large as the true value). Inference accuracy increases (albeit only slightly) after the correction of phasing errors. When the true phasing error parameter is known, inference accuracy is overall the best. Moreover, as shown in Figure 1 of main paper, without phasing error, accuracy can reach 95%. Thus, phasing error significantly affects the accuracy of parMix. It is highly desirable to run with data that has fewer phasing errors.

### Results on real and semi-simulated data

We now show results on real genetic data from the HapMap Project. Here, we use the genotypes data from the ASW population. We use the CEU and YRI populations from the HapMap Project as the ancestral populations, and the genotypes of the reference panel come from the 1000 Genomes Project phase 3 reference panel (http://bochet.gcc.biostat.washington.edu/beagle/1000_Genomes_phase3_v5a/). We use twenty ASW parental individuals (i.e., forty phased haplotypes) from 10 trio families in HapMap’s ASW population as the parents’ haplotypes. We use RFMix (Maples et al., 2013) to infer the ancestry of these parental haplotypes and use the inferred ancestry as the ground truth for comparison.

#### Real data

The HapMap ASW trio family has one child. We use the single child from each of the 10 trio families. They are *NA19702, NA19705, NA19828, NA19836, NA19902, NA19919, NA19918, NA20129, NA19983*, and *NA20128*. The HapMap Project only provides the unphased haplotypes of these 10 individuals. Thus, we apply Beagle 5.2 (Browning et al., 2021) to these haplotypes for phasing them. The reference panel used for phasing is also the ASW population from the 1000 Genomes Project.

#### Semi-simulated data

We are not aware of public genetic data on families with multiple children from an admixed population. In order to evaluate the performance of parMix on a family with multiple children, we simulate two additional children for each ASW trio with recombination rate $\rho =10^{-8}$ per bp. We then combine these simulated haplotypes to form (unphased) genotypes. This leads to unphased haplotypes from three children for each family. We apply Beagle 5.2 (Browning et al., 2021) to phase these children using the same reference panel as real data analysis. We thus obtain phased haplotypes from three children (one real and two simulated) per family.

**Figure 4:**
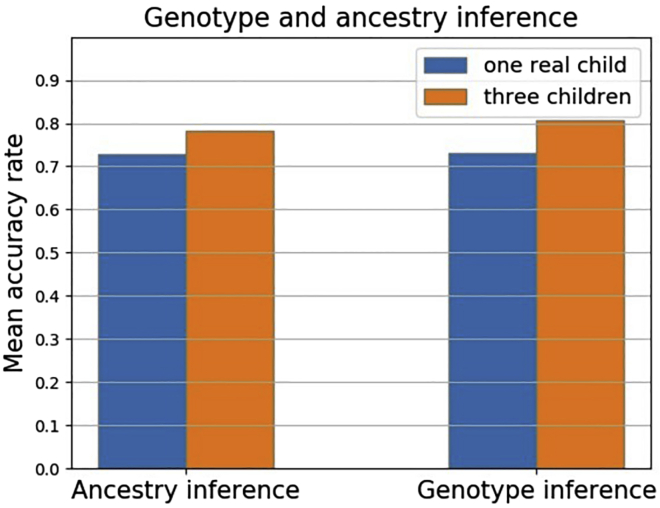
Ancestry and genotype inference results on real and semi-simulated data

**Figure 5:**
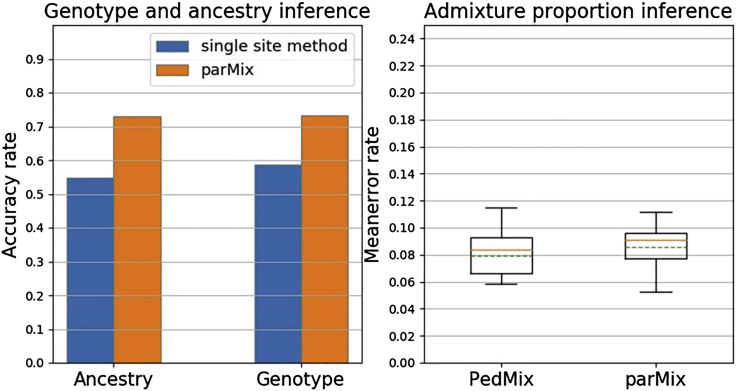
Comparison of parMix with other methods on real data Two cases: (i) parMix vs. single site method. (ii) parMix vs. PedMix.

We run parMix on the phased children’s haplotypes from one real child data and three children (one real and two semi-simulated). The phasing error rate is set as 0.0000002. As shown in Figure 4, the mean accuracy rate of parMix reaches 74% even with one child, and the highest mean accuracy rate exceeds 80% for the genotype inference with three children families after applying phasing error correction technique from PedMix (Pei et al., 2020). Moreover, as shown in Figure 5, we compare parMix with PedMix and the single site method using only one real child per family. Under the same settings, the accuracy of parMix is much higher than the single site method when inferring parental genotype and ancestry on real data. For admixture proportion inference, the mean error rate of parMix is slightly higher than PedMix for the one child data.

**Figure 6:**
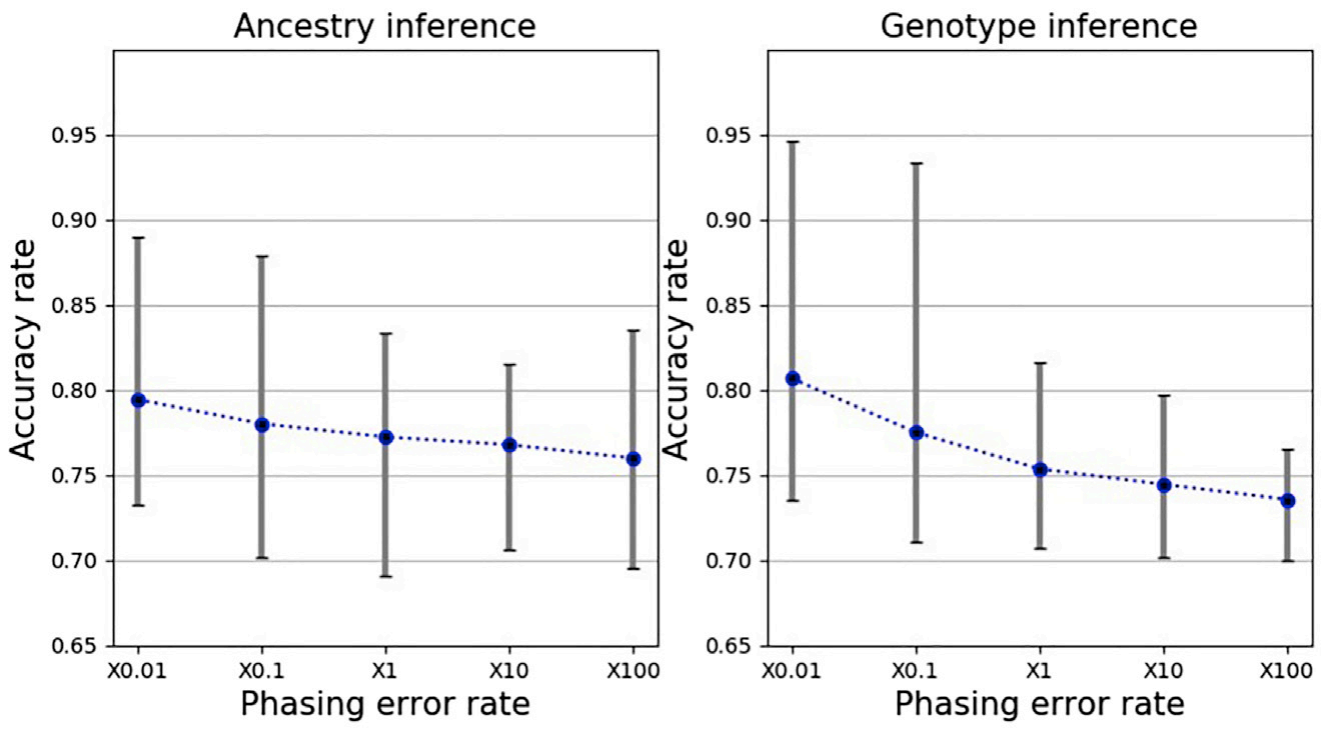
Impact of phasing error rates on the accuracy of ancestry inference and genotype calling for semi-simulated data

We now run parMix on the phased children’s haplotypes with different phasing error settings to call parental genotypes and ancestry. The default phasing error rate is 0.00002. To evaluate the impact of phasing error rate settings, we run parMix with phasing error rate varying from 0.01× default to 100× default. Three children per family are used. We also apply the phasing error correction technique in PedMix. As shown in Figure 6, as expected, the accuracy of parental genotype calling and ancestry inference are both ∼80%, which are similar to that of simulated data.

**Figure 7:**
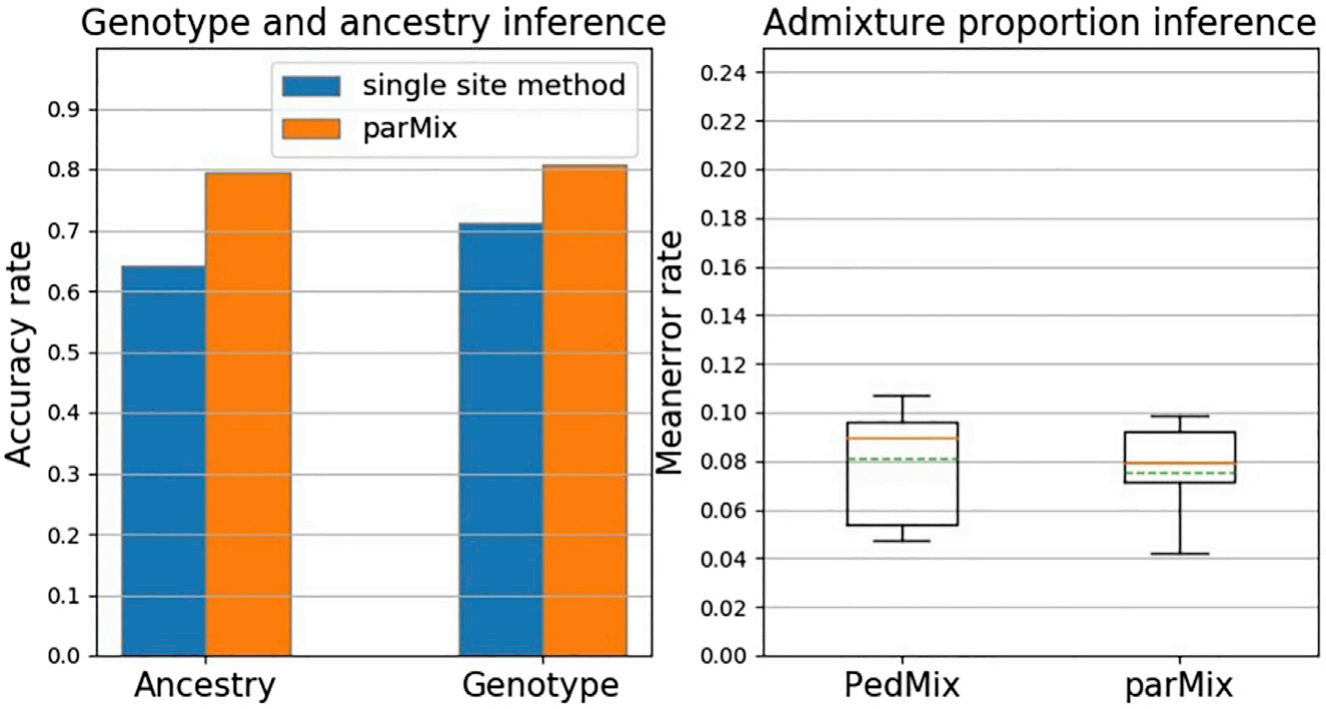
Comparison of parMix with other methods on semi-simulated data Two cases: (i) parMix vs. single site method. (ii) parMix vs. PedMix.

Finally, as shown in Figure 7, we also compare parMix with PedMix and the single site method using three semi-simulated children data. Under the same settings. The accuracy of parMix is still higher than that of the single site method when calling parental genotype and parental ancestry. For admixture proportion inference, the mean error rate of parMix is lower than PedMix.

### Running time of parMix

**Figure 8:**
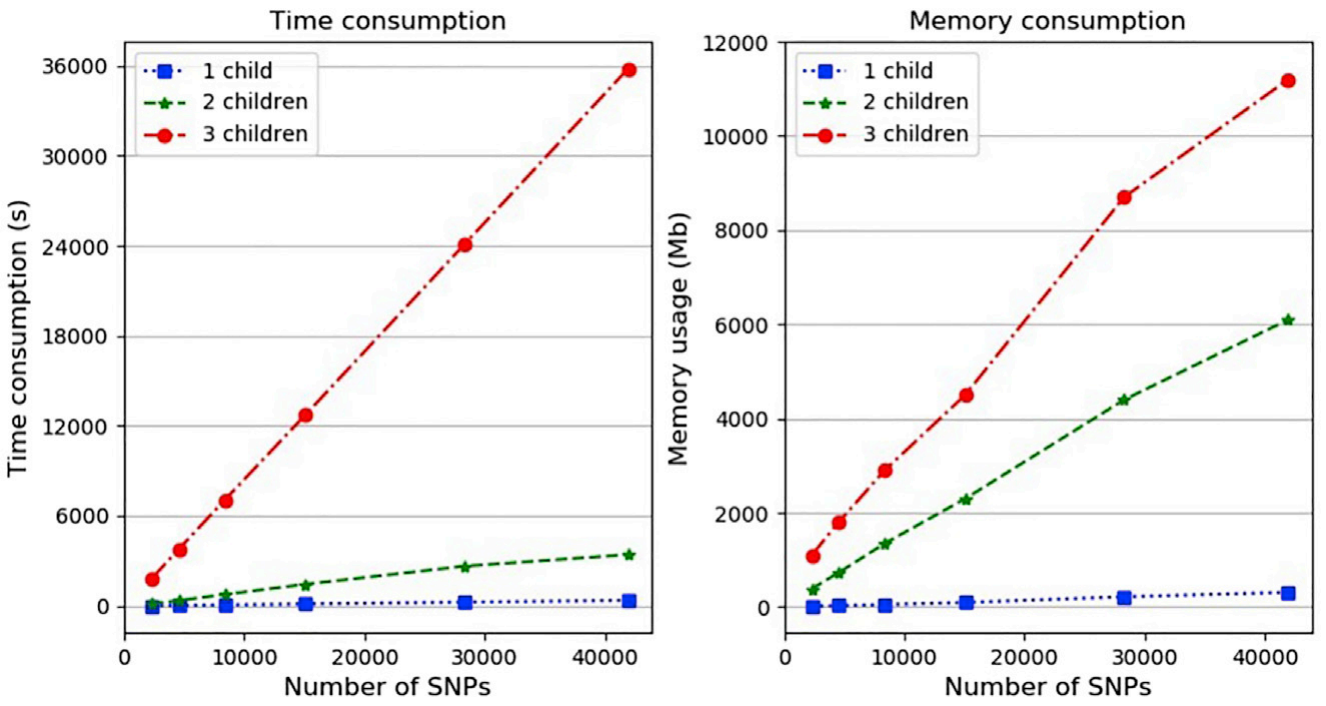
Running time and memory consumption of parMix One, two, and three children are used. Up to 40,000 SNPs are tested.

The running time of parMix mainly contains the time consumption and the memory consumption when running parMix. As shown in Figure 8, the time and memory consumption are influenced by the number of SNPs and the number of children in the experiment. For example, when there are three children, the stricter the phasing error rate is, the larger number of SNPs there is, which leads to higher running time and memory consumption. The memory consumption is mainly due to the forward and backward algorithm’s matrix, which increases fast when there are larger number of SNPs.

## Discussions and conclusion

In this paper, we present parMix, a method for joint inference of parental ancestry and genotypes from haplotypes of multiple children. Ancestral inference is clearly an important subject in genetics. Our method, parMix, is designed to work on a less-studied ancestral inference settings, where the members of a family with a number of children are admixed. While there are many methods (e.g., Structure) that can infer the ancestry of extant individuals (so-called chromosome painting). In some sense, the problem addressed by this paper is chromosome painting of parents based on genetic data of children. We are not aware of methods that infer ancestry of parents from genetic data of children. While this paper focuses on methodology development, we believe our method will potentially be useful for real genetics problems. This is partly because human beings are always interested in knowing something about ancestors.

Our results show that inference accuracy depends on the amount of genetic data available from children. With more children’s haplotypes, inference becomes more accurate (but also slower). Our results show that with three children, parMix can infer fairly accurate parental ancestry genotypes: even with phasing error, ancestry inference accuracy is ∼85%; without phasing error, it is ∼94%. Even with fewer children (say two or even one), parMix still can provide useful information about parents, although the variance of estimates is usually larger than that with more children.

There are few existing methods for calling genotypes and inferring ancestry of parents from genetic data of a small number of children. The closest existing method is PedMix (Pei et al., 2020) which only estimates parental admixture proportion. The inferred pointwise ancestry by parMix can be used to obtain an estimate of parental ancestry proportions. Compared to PedMix, the average mean error rate of parMix is lower than PedMix’s. This is likely due to that parMix infers admixture proportions from combined children’s genotypes, while PedMix processes each child’s genotype individually. However, it is worth mentioning that PedMix can trace back to more distant ancestors, such as grandparents. Also, the running time of PedMix is much lower than that of parMix.

Our real data and semi-simulated data analyses illustrate that parMix remains reasonably accurate when it is applied on the semi-simulated data. This indicates that parMix may be used to infer parental ancestry and genotypes for a multi-children family using real data. However, the input data of parMix, such as recombination rate (genetic map) and phasing error rate, can significantly affect the results. Therefore, it is important to use proper inputs when using parMix. However, different phasing methods may have different phasing error rates. The more accurate the phasing error rate is, the more accurate the result is. For example, when we use Beagle 3 (Browning and Browning, 2007) to phase the genotypes, the default phasing error rate gives the highest accuracy rate. But when we use Beagle 5.2 (Browning et al., 2021) (which produces more accurate haplotypes), we need to use a lower phasing error rate for getting the better result. Thus, the user should choose the phasing error parameter properly based on the data.

Indeed, phasing error apparently is the main technical challenge for parental ancestry/genotype inference. Our results show that without phasing error, the inference accuracy of parMix becomes very high. While current genetic data are prone to phasing error, we expect future technology development (e.g., long reads sequencing, and new phasing method) may greatly reduce phasing error in collected haplotypes and may make parMix more applicable.

The running time of parMix depends on the number of SNPs and the number of children *N*. The most time-consuming step is the inference of recombination and phasing vectors. There, the HMM enumerates configurations at each site, each with a 3N+4 bits binary vector. This leads to $2^{3N+4}$ configurations per site. When say N=10, the number of configurations becomes too large to enumerate. We apply the fast-computation algorithm for forward and backward algorithm, which is presented by PedMix (Pei et al., 2020), then the time complexity of parMix reduces to O(n(logn)). However, for families with large *N*, different inference approaches need to be used.

### Limitations of the study

The accuracy of parMix is significantly influenced by the phasing error in the given children’s haplotypes. Haplotypes are still not directly obtained from experiments. However, newer technologies (e.g., long reads sequencing) and tools are being developed. It is likely in the future, accurate haplotypes will be widely available. Moreover, parMix tends to work better with more children from a family. Existing public genetic data often only provide trio data, where there is only one child in the family. We expect genetic data from multiple children of a family will become available in the future.

## STAR★Methods

### Key resources table

**Table:** 

REAGENT or RESOURCE	SOURCE	IDENTIFIER
**Deposited data**		

Public data	HapMap	https://ftp.ncbi.nlm.nih.gov/hapmap

**Software and algorithms**		

parMix	This paper	https://github.com/biotoolscoders/parmix
Beagle	Brian Browning	https://faculty.washington.edu/browning/beagle
RFMix	Brian K. Maples	https://github.com/slowkoni/rfmix
PedMix	Jingwen Pei	https://github.com/yufengwudcs/PedMix

### Resource availability

#### Lead contact

Further information and requests for resources and materials should be directed to and will be fulfilled by the lead contact, Yufeng Wu (yufeng.wu@uconn.edu).

#### Materials availability

This study did not generate new unique materials.

### Method details

#### The high-level approach

Suppose we have phased haplotypes from *N* children in a family. Due to recombination, different segments of a child’s haplotype may originate from different haplotypes of a parent. Moreover, phasing error is often non-negligible in current genetic data. Therefore, it is not easy to determine from which parental haplotype a SNP allele of a child inherits from. The key observation is that heterozygous SNPs of a parent are very informative when there are multiple children. This is illustrated in the below Figure. For the ease of exposition, we first assume there is no phasing error. Then an entire child’s haplotype is from a single parent (possibly with recombination between two haplotypes of this parent). Further, suppose we know which children’s haplotypes are from the same parent (there are only a small number of such choices when *N* is small). In the below Figure, we consider a SNP site $s_{3}$ where the three children have alleles 0, 1 and 0 respectively. Then one can infer that the parent is heterozygous at $s_{3}$ (assuming the probability of genotyping errors is small). Note at the next SNP site $s_{4}$, children’s genotypes are 0, 1, and 1 respectively. Since the probability of recombination within one generation is usually low, a child likely inherits from the *same* parental haplotype at $s_{3}$ and $s_{4}$. So with high probability, there is a recombination between $s_{3}$ and $s_{4}$ when creating the third child.

When there is phasing error, when moving along a haplotype of a child, from which of two parents this haplotype inherits is no longer fixed. Assuming the phasing error rate is not too high, nearby parental heterozygous SNPs may still provide some hints about where recombination occurs since the probability of recombination is still low. Consider the right part of the below Figure. There are six haplotypes within the region from $s_{1}$ to $s_{9}$. Assuming no genotyping error and no recombination (both occur with a smaller probability than phasing error), there must be a phasing error somewhere. Further note that if we switch between the two haplotypes of child 1, we obtain two haplotypes that appear in the other two children. This is a strong indication of a phasing error. In practice, however, there is uncertainty for clearly calling phasing error and/or recombination due to factors such as genotyping errors, few numbers of children in data, and lack of heterozygous sites.

**Figure:**
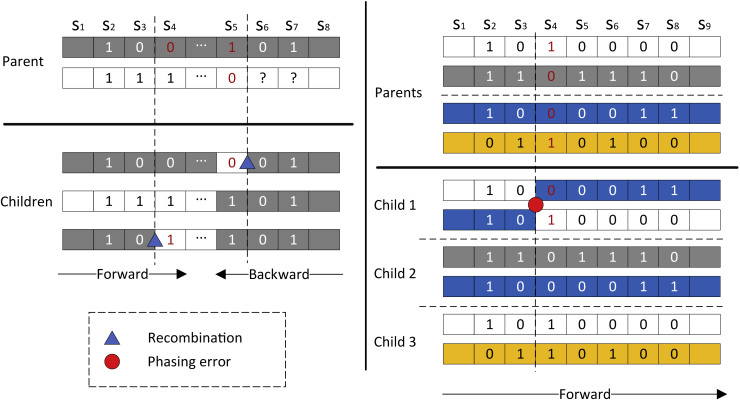
High-level approach (Left) No phasing error. Heterozygous SNP alleles of children provide hints on recombination (between $s_{3}$ and $s_{4}$ of third child and $s_{5}$ and $s_{6}$ of first child). (Right) With phasing errors. Phasing error (at the first child) can sometimes be detected by looking at haplotypes from all children.

To address the inherent uncertainty, we use hidden Markov model (HMM) as the main underlying probabilistic model. It is possible to use a single HMM to model all aspects of this inference problem: (i) recombination in parents, (ii) genotypes of parents, (iii) ancestry of parents, and (iv) phasing errors in children. However, our experience indicates that such an HMM is too complex and also leads to not very accurate inference. Instead, parMix takes a three-step procedure.

1. Infer (and then fix for later inference) locations of recombination and phasing errors from children’s haplotypes first.
2. Infer (and then fix for later inference) parental ancestry from children’s haplotypes.

#### Call parental genotypes

By dividing the inference into three steps, each step becomes more manageable. More importantly, this can lead to more accurate inference results because we first infer the aspects about which the data has more information. As described above, children’s haplotypes provide a strong indication about where recombination and phasing errors occur in children’s haplotypes. Thus, parMix infers recombination and phasing errors first.

#### Data and problem formulation

We consider a family of N≥1 children and two parents from an admixed population. Each individual in this family has admixed with M≥2 ancestral populations. In this paper, we assume M=2 (i.e., there are two ancestral populations). parMix can be easily extended to allow more than two ancestral populations, but will need more computational time. parMix takes the haplotypes $H_{k}$ for k={1,…,N} of the diploid children as input. Here, a haplotype is a binary vector of length *m*, and *m* is the number of SNPs (single nucleotide polymorphisms) within the haplotypes. We assume the haplotypes of children are genotyped and phased at SNP sites, possibly with phasing errors. Moreover, parMix takes population genetic information: (i) allele frequencies in each ancestral population are known for all SNPs, (ii) recombination distance between consecutive SNPs, and (iii) linkage disequilibrium (LD) in each ancestral population.

The primary goal of parMix is, for each SNP position, inferring the ancestry (which of two ancestral populations, say *A* and *B*) and genotypes of each parent. Since each parent is a diploid, there are four possible parental ancestries: AA, AB, BA, and BB, and four parental genotypes: 00, 01, 10, and 11.

Below Table lists the notations and parameters used in this paper.

**Table:** List of parameters and notations

Symbol	Description
*N*	The number of children
*M*	The number of reference populations
*T*	The number of SNPs
*t*	SNP site index
$d_{p}$	The physical distance between two SNPs
$d_{c}$	The genetic distance between two SNPs in centimorgan
$H_{t}$	The haplotype vector of children
$G_{t}$	The genotype vector of parent
$R_{t}$	The recombination vector of parent
$P_{t}$	The phasing vector of parent
$C_{t}$	The ancestry vector of parent

#### Hidden markov models

##### General HMM structure

Our inference is based on several structurally similar hidden Markov models. See below Figure for an illustration. All these HMMs have multiple states for each SNP site and there are transitions between any two states at consecutive SNPs. At each SNP, there are $2^{k}$ states, each corresponding to a distinct binary sequence (called configuration) of length-*k*. The meaning of configuration depends on the purpose of the inference and varies among HMMs. The key for these HMMs is the settings of transition and emission probabilities, which we will explain in more detail. Briefly, transition and emission probabilities are fully decided by the configurations involved and the provided population genetic information (e.g., allele frequencies and recombination fractions) based on standard genetic laws. That is, we don’t need to run the Baum-Welch algorithm to perform parameter estimation for the HMM. So after HMM is constructed, we can infer the states sequence using the standard posterior decoding algorithm from given data. The states directly correspond to what we want to infer. For example, suppose we want to infer parental genotypes in an HMM. The configurations at a site *s* of the HMM have bits that correspond to parental genotypes at *s*. To infer parental genotypes, we simply use posterior decoding to find the most likely configuration at *s*. In the following, we focus on how an HMM is constructed: (i) the meaning of the configuration bits, and (ii) transition and emission probabilities.

**Figure:**
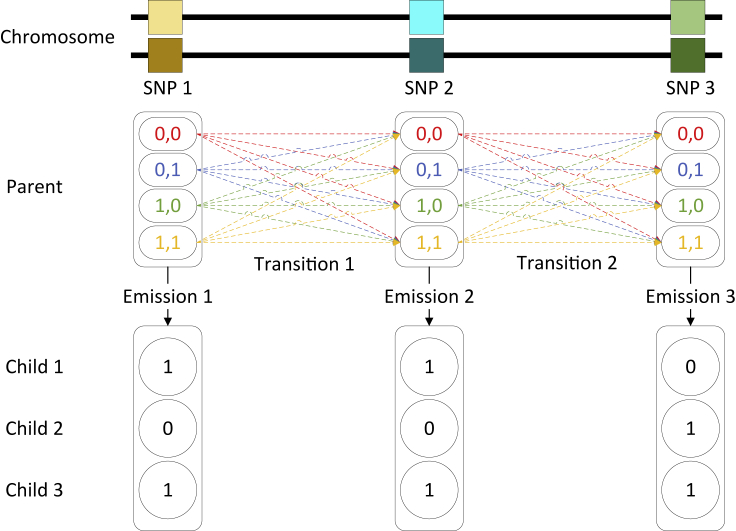
The calling method of the posterior decoding algorithm We start with the first fixed site $t_{1}$, and select$t_{2}$’s vector as (010) since it is same as the previous site’s. Then, the same strategy is used to site $t_{3}$, and it continues until it reaches the next fixed site $t_{5}$. In the situation that the first fixed site is not the first site of the sequence, like $t_{10}$. It will select$t_{9}$’s vector as (001) because it is same as the$t_{10}$’s. It continues until it reaches the first site $t_{7}$ of the sequence.

##### Inference of recombination and phasing errors

parMix first infers the locations of recombination events and phasing errors, which can then be used for later inference. The phasing error rate can be especially a problem for inference because in current data it is usually much higher than the recombination rate. For example, in human data, the average recombination rate is $10^{-8}$ per base pair per generation, while the phasing error rate can be $2\text{∗}10^{-5}$ per base pair in current data. Therefore, it is important to handle phasing errors in inference. We now describe an HMM model for inferring recombination and phasing errors in children’s haplotypes in a probabilistic way.

In this model, the observable data are the children’s haplotypes, which are represented by a length-2∗N binary haplotypes vector $H_{t}$ for each SNP site *t*. Let $AC_{t}$ denote a set containing all possible hidden states (configurations) at SNP site *t*, and each configuration $AC_{t}=\left(P_{t},R_{t},G_{t}\right)\in AC_{t}$ is a binary vector of length-(3N+4). Here, $P_{t}$ is the phasing vector of length *N* bits. For each child *i*, $P_{t}\left[i\right]=0$ (resp. 1) means child *i*’s allele is from paternal (resp. maternal) side at the SNP site *t*. $R_{t}$ is the recombination vector of length-2N. For child *i*, $R_{t}\left[i\right]$ (resp. $R_{t}\left[i+N\right]$) represents the first (resp. second) haplotype of child *i* is from which haplotype of their parent at site *t*. For example, when N=3, {0,0,0,1,1,0} represents that the first (resp. second) allele of the first two children are from the first (resp. second) haplotype of the paternal (resp. maternal) side at *t*, while both alleles of the third child are from the first allele of parents. $G_{t}$ is a binary vector of 2×2=4 bits, and it represents the genotypes of two parents at site *t*.

##### Transition and emission probabilities

We denote $p\left(AC_{t}|AC_{t-1}\right)$ as the transition probability from $AC_{t-1}$ at site t−1 to $AC_{t}$ at site *t*. Recall that $AC_{t}=\left(P_{t},R_{t},G_{t}\right)$. So $p\left(AC_{t}|AC_{t-1}\right)$ consists three parts, one for each vector in $AC_{t}$. (i) Recombination vector. We define $T_{j}$ as the transition probability of the recombination vector for $j_{th}$ child between sites t−1 and *t*. Let $d_{p}$ denote the number of base pairs between sites t−1 and *t*. We define $I_{t}^{R}=1$ if $R_{t}=R_{t-1}$, and 0 otherwise. Then, $T_{j}={\left(d_{p}\cdot r_{b}\right)}^{1-I_{t}^{R}}{\left(1-d_{p}\cdot r_{b}\right)}^{I_{t}^{R}}$, where $r_{b}$ is the recombination rate between two base pairs per individual per generation. If the given map is the centimorgan-based type, $T_{j}$ can also be easily calculated. (ii) Phasing vector. We denote $P_{j}$ as the transition probability of phasing vector between sites t−1 and *t* for the $j_{th}$ child. Similar to the transition probability of recombination vector, since the number of base pairs $d_{p}$ between two sites is known, We define $I_{t}^{P}=1$ if $P_{t}=P_{t-1}$, and 0 otherwise. Then, $P_{j}={\left(d_{p}\cdot p_{e}\right)}^{1-I_{t}^{P}}{\left(1-d_{p}\cdot p_{e}\right)}^{I_{t}^{P}}$, where $p_{e}$ is the phasing error rate between two base pairs per individual per generation. Here, since the phasing error rate $p_{e}$ is much larger than the recombination rate $r_{b}$, the transition probability of phasing vector $P_{j}$ may be larger than 1. If this happens, we set $P_{j}=1$. (iii) parental genotype vector. We denote *G* as the transition probability of the parental genotypes vector between two sites. Since there is uncertainty in parental ancestry, we simply assume any parental genotypes are of equal probability. We now combine all three parts to derive the overall transition probability between two configurations.

(Equation 1) $$p\left(AC_{t}|AC_{t-1}\right)=G^{f}\cdot G^{m}\cdot \prod_{j=1}^{N}P_{j}\cdot \prod_{j=1}^{N}T_{j}^{f}\cdot \prod_{j=1}^{N}T_{j}^{m}$$

where $T_{j}^{f}$ (and $T_{j}^{m}$) is the transition probability of recombination vector of the father (mother, respectively) for the $j_{th}$ child, and $G^{f}$ (and $G^{m}$) is the transition probability of the paternal (resp. maternal) genotype vector.

For emission probability, we consider a configuration $AC_{t}=\left(P_{t},R_{t},G_{t}\right)$ where children’s haplotypes $H_{t}$ at each site *t* are emitted. Note that $P_{t}$ and $R_{t}$ decides how alleles of parental alleles in $G_{t}$ are passed to $H_{t}$. Let $g_{e}$ be the genotyping error rate between children’s alleles. We define $I_{j}\left(AC_{t}\right)=1$ if $H_{t}^{j}$ is equal to the allele implies by $AC_{t}$ and 0 otherwise. Then the emission probability $p_{E}\left(AC_{t}\right)$ of configuration $AC_{t}$ is:

(Equation 2) $$p_{E}\left(AC_{t}\right)=\prod_{j=1}^{N}{\left(1-g_{e}\right)}^{I_{j}\left(AC_{t}\right)}g_{e}^{1-I_{j}\left(AC_{t}\right)}$$

##### Calling recombination and phasing vectors

After the construction of the first hidden Markov model, the posterior decoding algorithm is used to infer the configuration vector $AC_{t}$ at each locus. The posterior decoding algorithm provides a vectors sequence with the highest probability at each site, but there may be multiple maximum probability vectors at the same site. For example, in the three children model without phasing errors, the probability of recombination vector $R_{t}=\left(0,1,0\right)$ is same as the probability of vector $R_{t}=\left(1,0,1\right)$ since the initial probability distribution is equally distributed. Because recombination vector usually do not change greatly due to relatively low recombination rate, we apply a trimming procedure to trim the inferred vectors sequence.

As shown in the below Figure, we first find the sites that have only one configuration with the highest probability (in this example, the sites $t_{1}$ and $t_{5}$). Intuitively, the chosen recombination/phasing vectors at these sites are more likely to be correct than other sites. Then, based on these sites’ positions, we begin with the first fixed site ($t_{1}$) and trim the next site’s vector ($t_{2}$) with highest probability but in a different order until the next fixed site ($t_{5}$) is reached. Finally, we apply this method again for the first fixed site ($t_{10}$) but with the opposite direction until it reaches the first site of the sequence ($t_{7}$).

**Figure:**
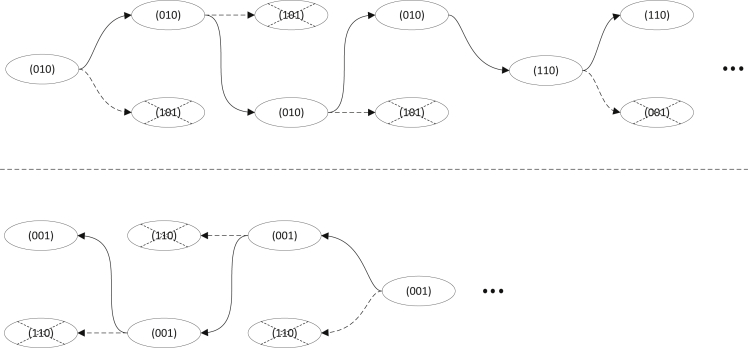
The general hidden Markov model that is used in this paper $2^{k}$ states at each column (SNP site). Each state has a distinct length-*k* binary string. States at two nearby sites are fully connected.

##### Example of the hidden Markov model

Below Figure shows an example of a family with two parents and three children. Each configuration consists of the phasing vector $\left(P_{1},P_{2},P_{3}\right)$, the recombination vector $\left(R_{1},R_{2},R_{3},R{\text{′}}_{1},R{\text{′}}_{2},R{\text{′}}_{3}\right)$, the genotype vector $(G_{1}^{f}$, $G_{2}^{f}$, $G_{1}^{m}$, $G_{2}^{m})$, and the haplotype vector $\left(H_{1}^{f},H_{1}^{m},H_{2}^{f},H_{2}^{m},H_{3}^{f},H_{3}^{m}\right)$. Suppose that $\left(P_{1},P_{2},P_{3}\right)=\left(0,0,1\right)$, $\left(R_{1},R_{2},R_{3},R{\text{′}}_{1},R{\text{′}}_{2},R{\text{′}}_{3}\right)$= (0,1,0,1,0,1), and $\left(G_{1}^{f},G_{2}^{f},G_{1}^{m},G_{2}^{m}\right)=\left(0,0,0,0\right)$. Then the configuration at this site *t*, denoted as $AC_{t}$, can be presented as $(P_{1}P_{2}P_{3}$, $R_{1}R_{2}R_{3}$, $R{\text{′}}_{1}R{\text{′}}_{2}R{\text{′}}_{3})$, $\left(G_{1}^{f}G_{2}^{f}G_{1}^{m}G_{2}^{m})\right)$ = (001,010101,0000).

**Figure:**
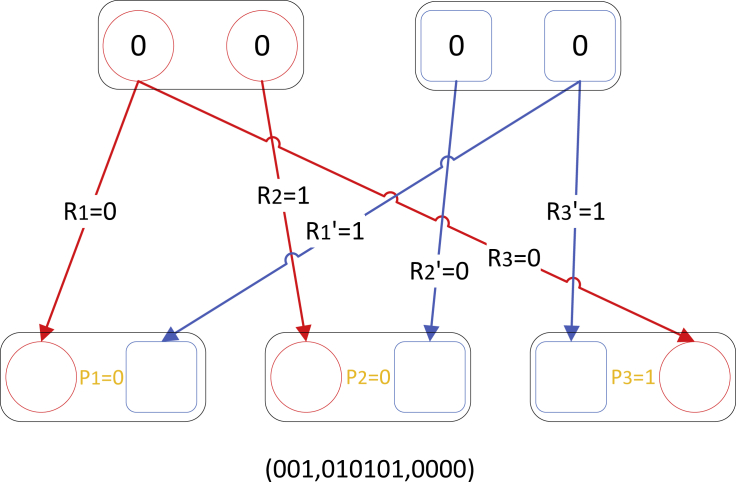
Example of configuration $AC_{t}$: two parents’ genotypes are both 00 The arrows with different colors show the paths of inheritance, and the first and third child’s genotypes can be traced to the first haplotype of the father and the second haplotype of the mother, but the second child’s genotype shows a different inheritance pattern. There is a phasing switch at the third child, which is denoted by $P_{3}=1$.

At two adjacent SNP sites, there are transitions between each pair of configurations. There are $2^{13}$ possible configurations at a site, and this leads to $2^{26}$ possible transitions between two adjacent SNPs. That is, if there are $N_{k}$ configurations at a site, the computational complexity for computing the transition probability is $O\left(N_{k}^{2}\right)$ at each site. In order to reduce the computational complexity, we use a divide and conquer algorithm which reduced the computational complexity to $O\left(N_{k}\cdot \text{log}N_{k}\right)$ for each site. Refer to Pei et al. (2020) for more details about this algorithm.

#### Inference of ancestry vector of parents

We now use the inferred phasing and recombination vectors to infer the ancestry of parents. We also use an HMM for this purpose. Here, the genotype vector $G_{t}^{f}$ $\left(G_{t}^{m}\right)$ is replaced by ancestry vector $C_{t}^{f}$ ($C_{t}^{m}$, respectively). $C_{t}^{f}$ $\left(C_{t}^{m}\right)$ is a binary vector of 1∗2=2 bits, and it represents the ancestral population of the parent. Therefore, if there are two reference populations, then this vector could be (0,0), (0,1), (1,0), or (1,1) which indicates that the parental ancestry can be both from population *A*, one of them came from population *A* and the other one is from population *B*, or both are from population *B*. Because the recombination and phasing vectors are fixed, the number of bits for the current model’s configuration is reduced to only four bits. Thus, inference of ancestry vectors is very efficient.

In detail, the $p\left({AC|}_{t}AC_{t-1}\right)$ is the transition probability from $AC_{t-1}$ at sit t−1 to $AC_{t}$ at site *t*. However, The difference is that, in this model, $AC_{t}=\left(C_{t}\right)$ without the vectors $P_{t}$ and $R_{t}$. We define $I_{t}^{C}=1$ if $C_{t-1}=C_{t}$, and 0 otherwise. Then $C_{j}=\left(I_{t}^{C}\times S^{g}\right)+f_{x}\times \left(1-S^{g}\right)$. $f_{x}$ is the admixture proportion for population *x* (*A* or *B*) depending on the value of $C_{t}$, and it can be set as 0.5 if the admixture proportion is unknown. Meanwhile, g is the number of generations since admixture, and we use g=10 by default. To simplify the notations, $p\left(AC_{t}|AC_{t-1}\right)$ can be re-defined as follow.

(Equation 3) $$C=p\left(C_{t}|C_{t-1}\right)=0.5+I_{t}^{C}\times 0.5\times S^{g}$$

where $I_{t}^{C}$ is 1 if $C_{t-1}=C_{t}$, and **-1** otherwise. Moreover, *S* is the **non-recombination** probability between two sites:

Therefore, the transition probability of this model can be written as follow.

(Equation 4) $$p\left(AC_{t}|AC_{t-1}\right)=\prod_{parent}C^{f}\times C^{m}$$

For the emission probability, let $f_{H_{j}}\left(C_{t}\right)$ be the allele frequency in the population specified by $\left(C_{t}\right)$ for the allele observed at site *t* of $j_{th}$ child’s haplotype, the emission probability of this hidden Markov model, denoted as $P_{E_{\left(H_{t}\right)}}\left(AC_{t}\right)$, can be defined as follows.

(Equation 5) $$P_{E_{H_{t}}}\left(AC_{t}\right)=\prod_{parent}\prod_{j}\left[\left(f_{H_{j}}\left(C_{t}\right)\cdot \left(1-g_{e}\right)\right)+\left(1-f_{H_{j}}\left(C_{t}\right)\right)\cdot g_{e}\right]$$

#### Inference of parental genotype vectors

Now that we have inferred recombination and phasing vectors as well as parental ancestry vectors, we can now infer genotype vector using another HMM. Here, the observed data includes children’s genotype vectors $H_{t}^{f}$ and $H_{t}^{m}$ at site *t*, along with parental ancestry vectors $C_{t}^{f}$ and $C_{t}^{m}$. The hidden states to infer are parental genotype vectors $G_{t}^{f}$ and $G_{t}^{m}$. Because parental ancestries along with recombination/phasing vectors are known, we can construct an HMM that incorporates both allele frequency and linkage disequilibrium in the transition probability. More specifically, consider two adjacent sites t−1 and *t*. Suppose the ancestry at t−1 is the same as that of *t* (say both from population *A*). Then the probability of observing a haplotype 00 in one parent is estimated to be the frequency of 00 with ancestral population *A* at these two sites. If t−1 has ancestry *A* but *t* has ancestry *B*, then the probability of having 00 is simply the allele frequency of 0 of population *B* (i.e., independent from the genotype at t−1).

Note that we have already inferred parental ancestry and recombination and phasing sequences in the previous steps. To infer parental genotypes, the naive way is to find the SNPs with the highest allele frequencies in the parents at each locus. However, this approach is not accurate because it only uses allele frequencies and ignores the linkage disequilibrium, and is only based on noisy inferred parental ancestry. Therefore we still need to use a hidden Markov model to infer the genotype vector, since the genotypes information of children is also included in the HMM model.

The emission probability of the third hidden Markov model is the same as the emission probability of the first hidden Markov model, which can be referred as Equation (2). But for the transition probabilities of the third model, the transition probability of $G_{t-1}$ to $G_{t}$ does not follow the equal distribution, and need to be defined based on the inferred ancestry vectors, $C_{t-1}$ and $C_{t}$.

We define $I_{t}^{G}=1$ if $C_{t-1}=C_{t}$, and 0 otherwise. Let *G* denotes the transition probability of two adjacent SNPs of one parent at site *t*. Then,

(Equation 6) $$G=p\left(G_{t}|G_{t-1}\right)={\left[f_{H_{t}}\left(C_{t}\right)\right]}^{1-I_{t}^{G}}\times {\left[ld_{\left(H_{t}|H_{t-1}\right)}\left(C_{t}|C_{t-1}\right)\right]}^{I_{t}^{G}}$$

where $f_{H_{t}}\left(C_{t}\right)$ is the allele frequency in the population specified by $\left(C_{t}\right)$ for the alleles configuration at site *t* of one parent’s haplotype, and $ld_{\left(H_{t}|H_{t-1}\right)}\left(C_{t}|C_{t-1}\right)$ is the linkage disequilibrium distribution in the population specified by $\left(C_{t-1}\right)$ and $\left(C_{t}\right)$ for the alleles configurations at sites t−1 and *t* of one parent’s haplotype.

The transition probabilities of the third hidden Markov model can be defined as:

(Equation 7) $$p\left(AC_{t}|AC_{t-1}\right)=\prod_{parent}G^{f}\times G^{m}$$

## Data Availability

• The data analysed in this paper are public published data, which can be downloaded from **HapMap** project’s website https://ftp.ncbi.nlm.nih.gov/hapmap. • The code of parMix is released on GitHub, which can be downloaded from https://github.com/biotoolscoders/parmix. • Any additional information and tools used in this paper are available from the lead contact upon request.
